# 固相萃取-液相色谱-串联质谱法同时测定污水中38种吲唑酰胺类合成大麻素及15种代谢产物

**DOI:** 10.3724/SP.J.1123.2024.11016

**Published:** 2025-10-08

**Authors:** Zhe YANG, Liwei JIANG, Yidi WU, Rui JIA, Jianxia LYU

**Affiliations:** 国家毒品实验室北京分中心，北京 100164; National Anti-Drug Laboratory Beijing Regional Center，Beijing 100164，China

**Keywords:** 固相萃取, 液相色谱-串联质谱, 合成大麻素, 代谢产物, 污水, solid phase extraction （SPE）, liquid chromatography-tandem mass spectrometry （LC-MS/MS）, synthetic cannabinoids （SCs）, metabolites, wastewater

## Abstract

合成大麻素被吸食后会以原体和代谢产物的形式随尿液和粪便排出，最终进入城市排污系统。为了对合成大麻素的滥用情况进行评估，本研究建立了固相萃取-液相色谱-三重四极杆质谱法（LC-MS/MS）同时对污水中38种吲唑酰胺类合成大麻素及15种代谢产物进行定性定量分析的方法。样品采用Oasis^Ⓡ ^MCX固相萃取柱进行前处理，在多反应监测（MRM）模式下，以0.1%甲酸水溶液和甲醇-乙腈（1∶1，v/v）混合溶液为流动相，采用岛津Shim-pack GIST-HP C18 AQ色谱柱（100 mm×2.1 mm，1.9 µm）进行分离，柱温40 ℃，流速0.4 mL/min，进样量10 µL。结果表明，采用该方法，38种合成大麻素及15种代谢产物可在11 min内被全部检测分析，在各自的线性范围内线性关系良好，相关系数（*r*）≥0.990 5，检出限为0.03～1.30 ng/L，定量限为0.11～4.30 ng/L，满足实际样品分析需求。采用10、50、200 ng/L 3个水平的38种合成大麻素及15种代谢产物混合标准溶液进行精密度试验，精密度（*n*=6）为2.1%～15.5%。以不含目标物的污水样品为基质样品，在10、50、200 ng/L 3个加标水平下进行加标回收试验，各待测物的加标回收率为61.2%～129.3%。本方法具有准确、快速、灵敏、分离效果好等优点，可以对生活污水中38种合成大麻素及15种代谢产物进行定性定量检测分析，从而为相关监测评估工作提供参考依据。

## Abstract (en)

Synthetic cannabinoids （SCs）， which are significantly more potent and efficacious than natural cannabis， are among the most widely abused new psychoactive substances. The emergence of the so-called first-generation SCs was followed by other SCs that eventually led to the development of eighth-generation indole/indazole amide-based SCs. Indeed， this entire category of SCs was added to the list of controlled substances on July 1， 2021. SCs can be disguised in various ways and are commonly sold in the form of electronic cigarette oil. SCs quickly enter the bloodstream through the lungs when smoked and are eventually excreted in the forms of precursors and metabolites in urine and feces following oxidation and metabolism， and finally enter the urban sewage system. SCs abuse can be studied and evaluated by analyzing SCs and their metabolites in municipal wastewater， thereby providing a practical reference. Therefore， methods for detecting SCs and their metabolites in wastewater need to be developed.In this study， a solid phase extraction （SPE）-liquid chromatography-tandem mass spectrometry （LC-MS/MS） method was developed for the simultaneous determination of 38 indazole amide SCs and 15 metabolites in wastewater. Samples were treated using Oasis^Ⓡ ^MCX SPE， and multiple reaction monitoring （MRM） mode was used with mobile phases comprising （A） 0.1% formic acid aqueous solution and （B） methanol-acetonitrile （1∶1， v/v）. The following gradient-elution conditions were used： 0-8 min， 55%A-15%A； 8-15 min， 15%A； 15-16 min， 15%A-55%A； 16-18 min， 55%A. Target analytes were separated using a SHIMADZU Shim-pack GIST-HP C18 AQ column （100 mm×2.1 mm， 1.9 µm） at a column temperature of 40 ℃ and a flow rate of 0.4 mL/min. The injection volume was 10 µL. The 38 SCs and 15 metabolites were identified within 11 min using MRM mode； these analytes exhibited good linear relationships and correlation coefficients （*r*） exceeding 0.990 5. The limits of detection were 0.03-1.30 ng/L， and the limits of quantification were 0.11-4.30 ng/L， respectively， which meet the requirements for analyzing SCs and their metabolites in actual samples. Precisions （*n*=6） were determined to be 2.1%–15.5% by spiking wastewater samples with 10， 50， and 200 ng/L of the 38 SCs and 15 metabolites. Recoveries of the 38 SCs and 15 metabolites were 61.2%-129.3% by spiking wastewater samples at low （10 ng/L）， medium （50 ng/L）， and high （200 ng/L） levels. Good performance was observed when real samples were analyzed. The developed method is accurate， rapid， sensitive， and effective for the determination of the 38 SCs and 15 metabolites in wastewater， thereby satisfying monitoring and evaluation requirements.

合成大麻素（SCs）是模拟大麻植物主要活性成分四氢大麻酚的化学作用而合成的一类化合物，通常具有更强大的危害性和成瘾性，能产生更为强烈的兴奋、致幻等效果^［1-4］^，且合成大麻素不依赖于大麻的种植，成本更低且容易获取，是目前世界上新精神活性物质中涵盖物质种类最多、滥用最为严重的家族^［5］^。自2006年出现第一代萘甲酰吲哚类合成大麻素起，目前已发展至第八代吲哚/吲唑酰胺类合成大麻素^［6，7］^。该类合成大麻素种类较新，自2021年7月1日我国整类列管合成大麻素类物质开始，相关案件数量逐渐增多^［7-15］^。

目前，合成大麻素类物质被伪装成各种形态，以电子烟油的形式贩卖是其中一种主要形式，其滥用方式为将合成大麻素类物质溶于电子烟油中吸食。吸毒人员吸食合成大麻素的过程中，该类物质经肺很快进入血液，经氧化、代谢后最终会以原体和代谢产物的形式随尿液和粪便排出，最终进入城市排污系统^［16-19］^。亲脂性较高的合成大麻素可以在肾内被肾小管被动重吸收，或者分布到脂肪组织，普遍以代谢物的形式通过尿液排泄，而亲水性较高的合成大麻素则容易通过尿液排出^［20，21］^。通过对城市生活污水中合成大麻素类物质及其代谢物进行检测分析，可以反映出不同合成大麻素的滥用情况，为实际执法提供参考依据。

污水分析法具有客观、普适性高、快速和简便等优点，近年来受到了越来越多的关注和应用^［22-24］^。目前固相萃取（SPE）是污水分析中最常见的前处理方法，污水样品经过固相萃取富集浓缩后，其灵敏度能达到ng/L^［25］^。有研究表明，与在线固相萃取相比，离线固相萃取对分析仪器的配制和灵敏度要求更低，在样品基质过于复杂、干扰峰难以分辨时更有优势^［22］^。

目前，国内外关于生活污水中毒品及其代谢物的检测主要有液相色谱-质谱法^［22-28］^和液相色谱-高分辨质谱法^［29，30］^。但是由于合成大麻素种类繁多、更新换代迅速、代谢产物种类多，检测分析方法并不能随着合成大麻素的更新换代及时更新。本研究拟采用固相萃取-液相色谱-三重四极杆质谱法（LC-MS/MS）同时检测生活污水中38种新型吲唑酰胺类合成大麻素及15种代谢产物，一次进样即可实现筛查和定量，从而更好地对合成大麻素类物质的毒情进行研判，对实际执法工作具有十分重要的意义。

## 1 实验部分

### 1.1 仪器、试剂与材料

液相色谱-三重四极杆质谱仪（LCMS-8060NX，日本岛津公司），液相色谱部分配备CBM-40控制器、SIL-40C X3自动进样器、CTO-40S柱温箱；质谱部分配备电喷雾离子源（ESI）。真空离心浓缩仪（CV400，北京吉艾姆科技有限公司），配备JM50低温冷阱、IKA Vacstar digital真空泵。固相萃取仪（Auto Pro SPE200），配备Auto Pro SPE200 V1.2.5工作站。

38种合成大麻素类物质及15种代谢产物、2种氘代同位素内标标准物质（5F-MDMB-PICA-D_5_作为38种合成大麻素氘代同位素内标；5F-MDMB-PICA metabolite 7-D_5_作为15种代谢产物氘代同位素内标）均为上海市刑事科学技术研究院与上海原思标物科技有限公司联合研制的标准物质，详细信息列于表1。甲醇、乙腈、乙酸铵、甲酸、氨水均为色谱纯，甲醇、乙腈、氨水均购自上海安谱实验科技股份有限公司，乙酸铵购自美国Fisher Scientific公司，甲酸购自北京百灵威科技有限公司；实验用水为超纯水，由Milli-Q Reference超纯水机制得。

**表1:** 38种合成大麻素、15种代谢物和2种氘代同位素内标的信息

No.	Compound	Retention time/min	Precursor ion （*m/z*）	Product ions （*m/z*）（Collision energies/eV）
1	ADB-BUTINACA *N*-butanoic acid metabolite	1.75	361.2	231.0^*^ （‒26）， 145.0 （‒40）， 213.0 （‒32）， 316.0 （‒15）
2	*N*-（1-amino-3-methyl-1-oxobutan-2-yl）-1-（4-cyanobutyl）-1*H*-indazole-3-carboxamide	1.76	342.42	226.0^*^ （‒26）， 145.0 （‒43）
3	*N*-（1-amino-3-methyl-1-oxobutan-2-yl）-1-（4-fluorobutyl）-1*H*-indazole-3-carboxamide	2.27	335.1	219.1^*^ （‒24）， 145.0 （‒41）， 177.0 （‒35）
4	methyl 2-（1*H*-indole-3-carbonylamino）-3，3-dimethylbutanoate	2.50	289.3	144.0^*^ （‒19）， 116.0 （‒44）
5	5F-AB-PINACA	2.63	349.2	233.1^*^ （‒24）， 304.2 （‒16）， 213.1 （‒32）
6	5F-MDMB-PICA metabolite 8	2.67	361.4	216.0^*^ （‒16）， 144.0 （‒39）
7	methyl 2-（1*H*-indazole-3-carboxamido）-3，3-dimethylbutanoate	2.70	290.4	145.0^*^ （‒29）， 117.0 （‒46）
8	5F-MDMB-PICA metabolite 9	2.80	393.5	248.0^*^ （‒20）， 160.0 （‒38）， 132.0 （‒53）
9	AB-FUBINACA	2.89	369.2	109.0^*^ （‒44）， 324.1 （‒16）， 253.0 （‒24）
10	4F-MDMB-BUTICA butanoic acid metabolite	2.89	349.5	218.0^*^ （‒17）， 144.0（‒38）， 116.0 （‒55）
11	5F-MDMB-PICA metabolite 2	2.96	375.5	230.0^*^ （‒19）， 144.0 （‒34）， 116.0 （‒53）
12	PX-2	3.00	397.2	233.1^*^ （‒25）， 213.0 （‒33）， 145.0 （‒46）
13	5F-ADB-PINACA	3.08	363.2	233.1^*^ （‒24）， 318.2 （‒16）， 177.0 （‒36）
14	ADB-BICA	3.08	364.2	347.2^*^ （‒10）， 234.1 （‒19）
15	5Cl-AB-PINACA	3.16	365.2	249.0^*^ （‒25）， 145.0 （‒43）， 213.0 （‒33）
16	JWH-073 *N*-butanoic acid metabolite	3.16	358.4	155.0^*^ （‒22）， 144.0 （‒32）， 127.0 （‒46）
17	5F MDMB-PICA metabolite 7	3.21	363.5	232.0^*^ （‒19）， 116.0 （‒53）， 144.0 （‒39）
18	ADB-BINACA	3.32	365.2	235.1^*^ （‒25）， 320.2 （‒15）， 348.2 （‒10）
19	4CN-MDMB-BUTINACA	3.33	371.2	226.0^*^ （‒28）， 145.0 （‒44）
20	ADB-FUBINACA	3.33	383.2	338.2^*^ （‒16）， 253.1 （‒26）
21	JWH-018 *N*-pentanoic acid metabolite	3.35	372.15	155.0^*^ （‒23）， 127.0 （‒46）， 244.0 （‒24）
22	AMB-FUBINACA metabolite	3.36	370.4	109.0^*^ （‒39）， 253.0 （‒21）
23	AB-PINACA	3.48	331.2	215.1^*^ （‒24）， 286.2 （‒15）， 314.2 （‒10）
24	ADB-BUTINACA	3.48	331.2	286.2^*^ （‒15）， 201.1 （‒27）， 145.0 （‒41），
25	4F-MDMB-BUTINACA butanoic acid metabolite	3.49	350.5	219.0^*^ （‒22）， 145.0 （‒41）， 177.0 （‒33）
26	4CN-CUMYL-BUTINACA	3.50	361.2	226.1^*^ （‒22）， 243.1 （‒12）， 145.0 （‒42）
27	ADB-4en-PINACA	3.54	343.2	213.1^*^ （‒24）， 298.2 （‒16）， 171.1 （‒39）
28	4F-MMB-BUTINACA	3.54	350.4	219.0^*^ （‒24）， 145.0 （‒39）， 177.0 （‒33）
29	UR-144 *N*-pentanoic acid metabolite	3.81	342.2	125.0^*^（‒20）， 144.0 （‒35） 244.0 （‒22）
30	CUMYL-THPINACA	3.96	378.2	243.1^*^ （‒22）， 260.1 （‒12）， 119.1 （‒25）
31	ADB-PINACA	3.97	345.2	215.1^*^ （‒26）， 300.2 （‒15）， 328.2 （‒10）
32	MDMB-4en-PINACA butanoic acid metabolite	3.97	344.5	213.0^*^（‒21）， 171.0 （‒34）， 145.0 （‒37）
33	AB-CHMINACA	4.02	357.0	241.0^*^（‒26）， 312.0 （‒17）
34	AMB-FUBINACA	4.09	384.2	109.0^* ^（‒44）， 253.1 （‒23）， 324.2 （‒16）
35	1-（4-fluorobutyl）-*N*-（2-phenylpropan-2-yl）- 1*H*-indazole-3-carboxamide	4.15	354.4	219.1^* ^（‒21）， 119.0 （‒24）， 145.05 （‒41）
36	4F-ABUTINACA *N*-（4-hydroxybutyl） metabolite	4.29	368.5	135.0^*^（‒22）， 107.0 （‒45）， 216.0 （‒15）
37	5F-CUMYL-PINACA	4.53	368.2	233.1^*^ （‒20）， 250.1（‒11）， 213.1（‒30）
38	EMB-FUBINACA	4.58	398.2	109.0^*^ （‒48）， 324.2 （‒17）， 352.1 （‒13）
39	5F-EDMB-PINACA	4.84	392.2	233.1^*^（‒25）， 318.2 （‒17）， 213.1 （‒33）
40	5Cl-ADB	4.98	394.9	250.0^*^ （‒25）， 214.0 （‒33）， 145.05 （‒42）
41	MDMB-BUTINACA	4.98	346.2	201.1^* ^（‒24）， 286.2 （‒16）， 145.0 （‒39）
42	MDMB-4en-PINACA	5.02	358.2	213.1^*^ （‒24）， 298.2 （‒16）， 145.0 （‒41）
43	5F-MN-18	5.06	376.2	233.1^*^ （‒17）， 145.0 （‒38）， 213.1 （‒28）
44	5Cl-CUMYL-PINACA	5.27	384.85	250.0^*^ （‒22）， 214.0 （‒31）， 145.0 （‒42）， 119.0 （‒25）
45	4F-ABUTINACA	6.10	370.2	135.1^*^ （‒20）， 93.1 （‒51）， 107.1 （‒44）
46	EDMB-PINACA	6.70	374.2	215.1^*^ （‒26）， 300.3 （‒17）， 145.0 （‒42）， 328.2 （‒13）
47	5F-APINACA	6.89	384.2	135.1^* ^（‒20）， 107.1 （‒45）
48	MDMB-CHMINACA	7.06	386.2	241.1^*^ （‒25）， 326.2 （‒17）， 354.2 （‒13）
49	MN-18	7.26	358.2	215.15^*^ （‒19）， 145.0 （‒34）
50	FUB-APINACA	7.60	404.2	135.1^*^ （‒21）， 107.1 （‒49）
51	5Cl-APINACA	8.59	400.2	135.1^*^ （‒23）， 107.1 （‒48）
52	APINACA	10.05	366.2	135.1^*^ （‒22）， 107.1 （‒42）
53	ACHMINACA	10.83	392.3	135.1^*^ （‒23）， 93.1 （‒53）， 107.1 （‒50）
54	5F-MDMB-PICA metabolite 7-D_5_	3.20	368.5	237.0^*^ （‒19）， 149.0 （‒40）
55	5F-MDMB-PICA-D_5_	3.88	382.5	237.0^*^ （‒17）， 149.0 （‒41）， 121.0 （‒54）

* Quantitative ion.

固相萃取柱Oasis^Ⓡ ^HLB柱、Oasis^Ⓡ ^MAX柱、Oasis^Ⓡ ^MCX柱规格均为60 mg/3 mL，购自美国Waters公司。

实验用污水采集自某省某污水处理厂进水，采样使用自动采样器，采集方式为每隔2 h采集一次，一天采集12次，连续采样一周。采集的水样混合在一起装入聚丙烯瓶中。

### 1.2 标准溶液的配制

称取各标准物质，分别用甲醇溶解后定容于10 mL容量瓶中，得到38种合成大麻素类物质及15种代谢产物质量浓度均为100 µg/mL的标准储备液。量取各目标物标准储备液适量，用甲醇稀释，定容于20 mL容量瓶中，配制成53种目标物质量浓度均为500 ng/mL的混合标准溶液，待用。

分别称取2种氘代同位素内标，用甲醇溶解后定容于10 mL容量瓶中，得到2种氘代同位素内标质量浓度均为100 µg/mL的内标标准储备液。量取各目标物标准储备液适量，用甲醇稀释，配制成2种氘代同位素内标质量浓度均为50 ng/mL的混合内标标准溶液，待用。

### 1.3 样品前处理

样品使用抽滤装置过滤后，量取10 mL于具盖离心管中，加入100 µL质量分数为30%的乙酸铵溶液，振荡混匀后，加入50 ng/mL混合内标标准溶液10 µL，振荡混匀。样品经上述处理后，以4 mL/min流速加载至Oasis^Ⓡ ^MCX固相萃取柱中，纯水4 mL淋洗（淋洗流速2 mL/min），再用4 mL 5%氨水甲醇溶液洗脱（洗脱流速1 mL/min），收集洗脱液。使用真空离心浓缩仪于50 ℃将洗脱液浓缩至干，加入甲醇100 µL复溶，振荡混匀后进样分析。

### 1.4 仪器条件

色谱和质谱条件见文献［^31^］，其中进样量为10 µL，其他质谱参数见表1。

## 2 结果与讨论

### 2.1 质谱条件的优化

使用合成大麻素混合标准溶液和混合氘代同位素内标溶液，分别在正离子模式和负离子模式下进行质谱条件优化。结果表明，38种合成大麻素和15种代谢产物，以及2种氘代同位素内标在ESI^+^模式下均可产生较高强度［M+H］^+^准分子离子峰，在此基础上选择响应值高、特征性强的子离子作为定性、定量离子。优化后的各化合物的质谱参数详见表1。

### 2.2 色谱条件的优化

本实验考察了分别以水和0.1%甲酸水溶液为水相时的分析结果，以及分别以甲醇、乙腈和甲醇-乙腈（1∶1，v/v）混合溶液为有机相时的分析结果。具体见参考文献［^31^］。最终选择0.1%甲酸水溶液为水相，甲醇-乙腈（1∶1，v/v）混合溶液为有机相。

由于不同结构的合成大麻素及代谢产物的极性存在较大差异^［32］^，为了保证所有物质均可以在合适的时间快速出峰，优先采用梯度洗脱的方式进行色谱分析。在该条件下，38种合成大麻素及15种代谢产物的色谱峰峰形较好（见图1）。

**图1:**
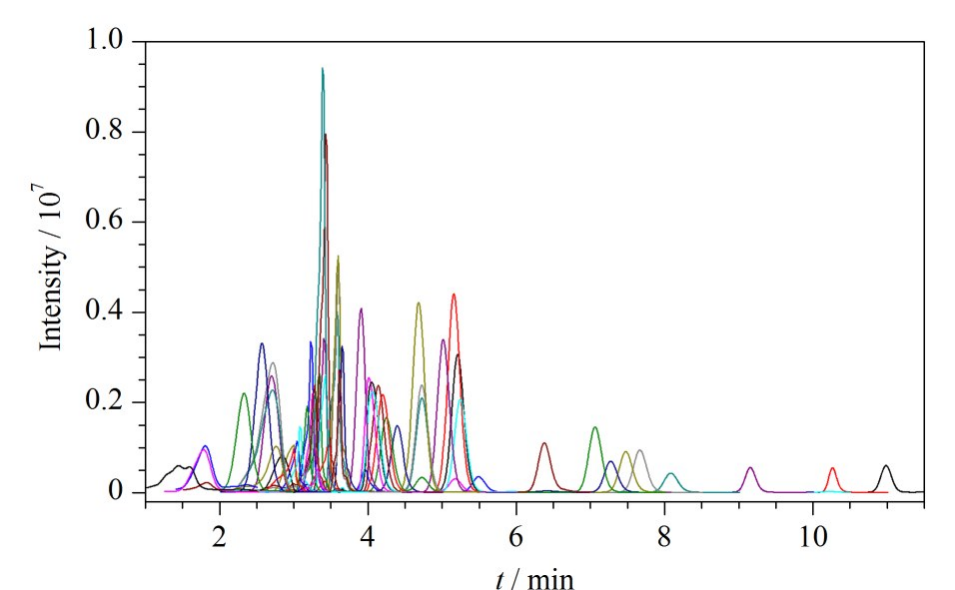
38种吲唑酰胺类合成大麻素和15种代谢产物的色谱图

### 2.3 样品前处理的优化

#### 2.3.1 固相萃取柱的选择

取不含目标物的污水水样10 mL，加入100 µL质量分数为30%的乙酸铵溶液，振荡混匀后，添加38种合成大麻素及15种代谢产物混合标准溶液，振荡混匀，配制成实验用水样。

Oasis^Ⓡ ^HLB柱和Oasis^Ⓡ ^MCX柱是环境水样前处理中较为广泛使用的两种固相萃取柱^［33］^，且被广泛应用于污水中常见毒品和新精神活性物质的富集。此外，本实验15种代谢产物大部分为酸性化合物。因此，实验分别考察了Oasis^Ⓡ ^HLB、Oasis^Ⓡ ^MCX和Oasis^Ⓡ ^MAX共3种固相萃取柱对水样中合成大麻素及代谢产物的萃取富集效果。结果显示，使用Oasis^Ⓡ ^MCX柱时，有17种目标物的响应略强于Oasis^Ⓡ ^HLB柱和Oasis^Ⓡ ^MAX柱（见图2）；而另外36种化合物的响应，在使用3种不同SPE柱时没有明显差别。本实验室在检测污水中常规毒品时，使用的为Oasis^Ⓡ ^MCX柱。因此综合考虑，最终选择Oasis^Ⓡ ^MCX柱对污水中的目标物进行萃取富集。

**图2:**
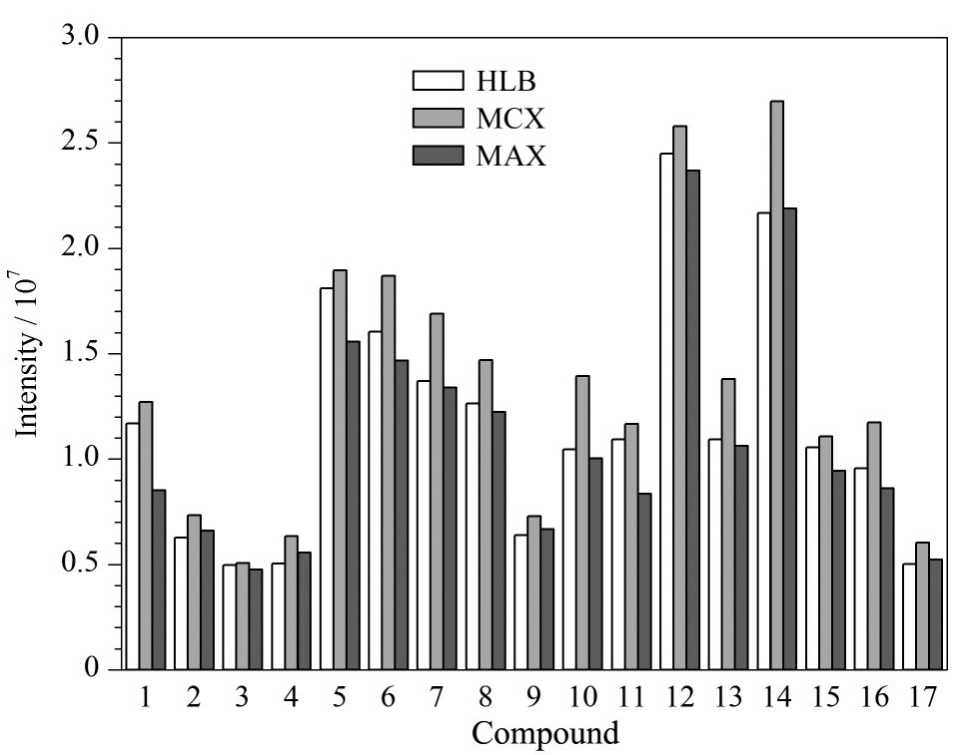
使用不同固相萃取柱时17种化合物的响应 Compounds 1-17： 1. 5F-APINACA； 2. AB-CHMINACA； 3. AB-FUBINACA； 4. ADB-PINACA； 5. 4CN-MDMB-BUTINACA； 6. 1-（4-fluorobutyl）-*N*-（2-phenylpropan-2-yl）-1*H*-indazole-3-carboxamide； 7. 5F-EDMB-PINACA； 8. EMB-FUBINACA； 9. MN-18； 10. CUMYL-THPINACA； 11. MDMB-CHMINACA； 12. EMB-FUBINACA； 13. AMB-FUBINACA； 14. 5F-CUMYL-PINACA； 15. UR-144 *N*-pentanoic acid metabolite； 16. 4F-ABUTINACA *N*-（4-hydroxybutyl） metabolite； 17. MDMB-4en-PINACA butanoic acid metabolite.

#### 2.3.2 淋洗溶剂的选择

上样后的淋洗步骤主要是为了去除吸附在固相萃取柱上的干扰物^［34］^。本实验分别考察了含不同体积分数（0、1%、2%、5%、8%、10%、20%）甲醇的水溶液的淋洗效果。结果表明，随着甲醇比例的增加，合成大麻素的响应没有明显变化趋势，而部分代谢物的响应随之降低。当甲醇比例增加时，ADB-BUTINACA *N*-butanoic acid metabolite、MDMB-4en-PINACA butanoic acid metabolite、JWH-073 *N*-butanoic acid metabolite、5-fluoro MDMB-PICA metabolite 7、5F-MDMB-PICA metabolite 8、4F-MDMB-BUTICA butanoic acid metabolite和AMB-FUBINACA metabolite等7种代谢产物响应变化最明显，响应明显降低（见图3）。实验结果表明，淋洗溶剂中加入甲醇后，部分代谢物会被淋洗进废液中，造成目标物的响应强度变低。因此，最终选择水作为淋洗溶剂。

**图3:**
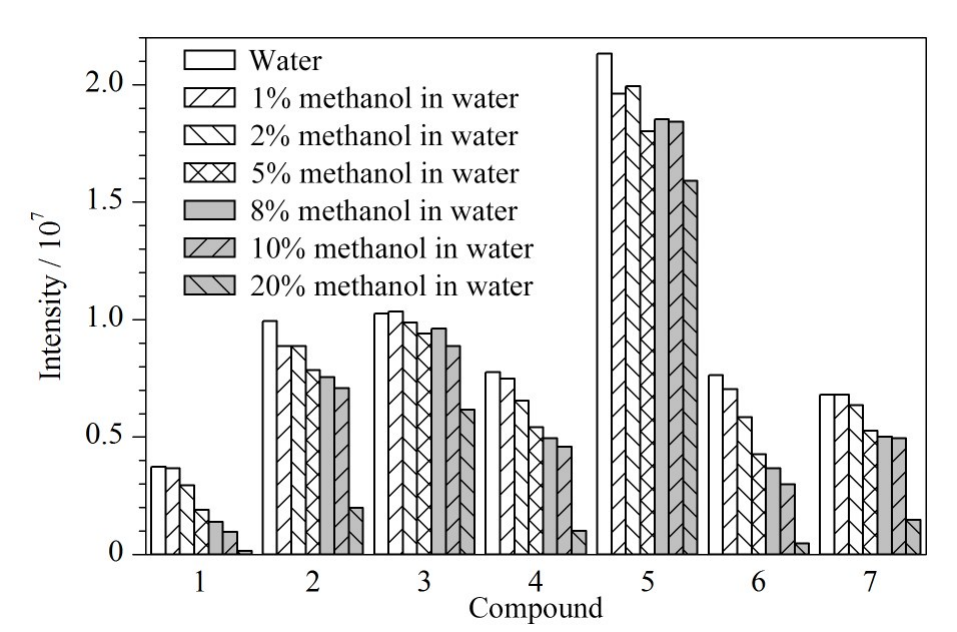
使用不同淋洗溶剂时7种代谢产物的响应 Compounds 1-7： 1. ADB-BUTINACA *N*-butanoic acid metabolite； 2. MDMB-4en-PINACA butanoic acid metabolite； 3. JWH-073 *N*-butanoic acid metabolite； 4. 5F MDMB-PICA metabolite 7； 5. 5F-MDMB-PICA metabolite 8； 6. 4F-MDMB-BUTICA butanoic acid metabolite； 7. AMB-FUBINACA metabolite.

#### 2.3.3 洗脱溶剂的选择

本实验考察了甲醇和5%氨水甲醇对目标物的洗脱效果。结果表明，与用甲醇洗脱相比，用5%氨水甲醇洗脱时，有23种目标物的响应增加（见图4），其中包括20种合成大麻素和3种代谢物，这说明在使用甲醇进行洗脱时，该23种目标物没有被完全洗脱。因此，最终选择5%氨水甲醇作为洗脱溶剂。

**图4:**
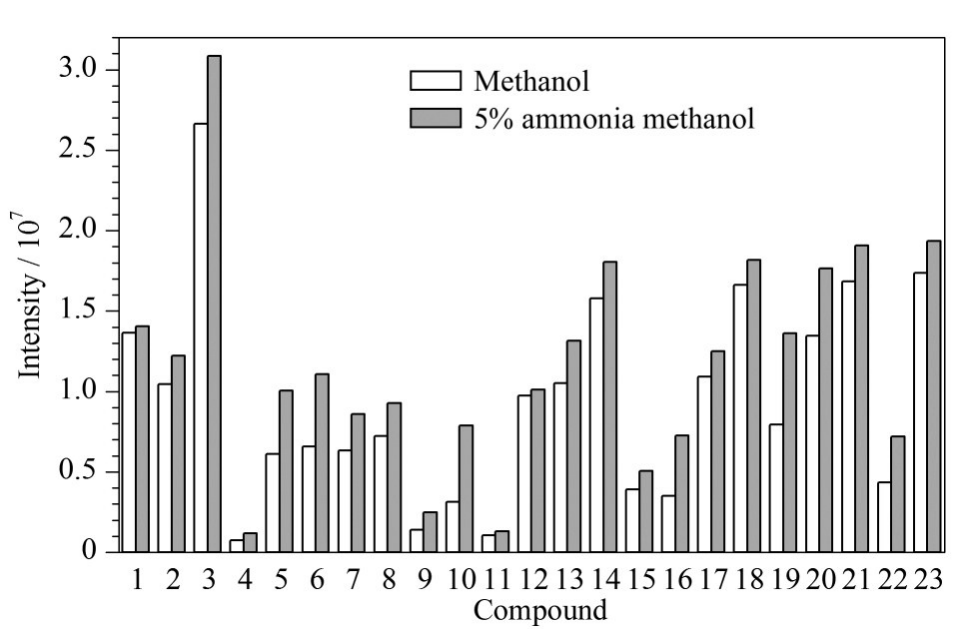
使用不同洗脱溶剂时20种合成大麻素和3种代谢产物的响应 Compounds 1-23： 1. AB-CHMINACA； 2. ADB-PINACA； 3. 4CN-MDMB-BUTINACA； 4. 5Cl-ADB； 5. 4F-MMB-BUTINACA； 6. 5F-EDMB-PINACA； 7. 5F-MN-18； 8. EMB-FUBINACA； 9. EDMB-PINACA； 10. MDMB-BUTINACA； 11. 5Cl-CUMYL-PINACA； 12. 5F-ADB-PINACA； 13. CUMYL-THPINACA； 14. ADB-BUTINACA； 15. 4F-ABUTINACA； 16. MDMB-4en-PINACA； 17. ADB-4en-PINACA； 18. AB-PINACA； 19. AMB-FUBINACA. 20. 5F-CUMYL-PINACA； 21. 4F-ABUTINACA *N*-（4-hydroxybutyl） metabolite； 22. 4F-MDMB-BUTINACA butanoic acid metabolite； 23. methyl 2-（1*H*-indole-3-carbonylamino）-3，3-dimethylbutanoate.

### 2.4 基质效应的评价

实验对基质效应进行了考察，按照基质效应＝空白基质溶液中待测物的响应值／空白纯水溶液中待测物的响应值×100%^［35］^。当基质效应低于80%时为基质抑制效应，当基质效应高于120%时为基质增强效应，当基质效应为80%~120%时，认为没有基质效应。

取水和不含目标物的污水样品按照1.3节方法处理，使用38种合成大麻素及15种代谢产物混合标准溶液进行复溶，分析10、50、200 ng/L（固相萃取前的质量浓度）3个水平下的基质效应。实验结果表明：（1）在10 ng/L添加水平下，6种目标物的基质效应为121.5%～148.1%，表现为基质增强效应；10种目标物的基质效应为43.6%～79.6%，表现为基质抑制效应；37种目标物的基质效应为83.2%～113.9%，没有表现出明显的基质效应。（2）在50 ng/L添加水平下，7种目标物的基质效应为121.1%～138.0%，表现为基质增强效应；7种目标物的基质效应为62.8%～76.8%，表现为基质抑制效应；39种目标物的基质效应为82.5%～117.4%，没有表现出明显的基质效应。（3）在200 ng/L添加水平下，2种目标物的基质效应为120.7%～128.3%，表现为基质增强效应；3种目标物的基质效应为78.3%～78.7%，表现为基质抑制效应；48种目标物的基质效应为83.2%～113.4%，没有表现出明显的基质效应（见表2）。目前常用的减少基质效应影响的方法有同位素内标、减少进样量、基质匹配标准曲线等。为了使定量更加准确、可靠，本实验采用同位素内标法进行定量，从而减少基质效应的影响。

**表2:** 38种合成大麻素和15种代谢物的线性范围、检出限、定量限、基质效应、回收率和精密度

No.	Compound	Linear range/（ng/L）	LOD/（ng/L）	LOQ/（ng/L）	Matrix effects/%			Recoveries/% （*n*=6）			RSDs/% （*n*=6）		
					10 ng/L	50 ng/L	200 ng/L	10 ng/L	50 ng/L	200 ng/L	10 ng/L	50 ng/L	200 ng/L
1	ADB-BUTINACA *N*-butanoic acid metabolite	5-250	1.30	4.30	75.4	111.0	106.9	69.1	85.8	90.6	6.8	7.1	7.2
2	*N*-（1-amino-3-methyl-1-oxobutan-2-yl）-1-（4-cyanobutyl）-1*H*-indazole-3-carboxamide	1-250	0.05	0.16	89.0	110.3	106.1	71.9	74.5	81.0	5.0	9.8	11.8
3	*N*-（1-amino-3-methyl-1-oxobutan-2-yl）-1-（4-fluorobutyl）-1*H*-indazole-3-carboxamide	5-250	0.24	0.79	96.9	121.1	106.3	77.5	75.7	74.7	12.8	6.4	14.2
4	methyl 2-（1*H*-indole-3-carbonylamino）-3，3-dimethylbutanoate	5-250	0.43	1.42	95.8	88.3	105.1	62.0	89.2	75.8	14.2	7.8	11.4
5	5F-AB-PINACA	1-250	0.22	0.73	147.2	127.3	112.1	116.4	75.1	81.6	12.8	10.6	12.4
6	5F-MDMB-PICA metabolite 8	5-250	0.68	2.25	143.3	84.6	103.0	121.1	100.4	104.3	12.8	7.9	7.1
7	methyl 2-（1*H*-indazole-3-carboxamido）-3，3-dimethylbutanoate	5-250	0.39	1.27	148.1	123.3	104.3	103.0	83.2	81.1	12.6	10.5	8.6
8	5F-MDMB-PICA metabolite 9	5-250	0.13	0.43	142.3	88.9	105.6	87.1	124.5	127.5	8.1	6.2	7.9
9	AB-FUBINACA	1-250	0.13	0.42	72.1	114.5	108.1	70.6	80.5	90.7	8.0	9.3	14.5
10	4F-MDMB-BUTICA butanoic acid metabolite	5-250	0.14	0.46	65.2	101.3	102.0	61.8	98.2	105.8	2.5	4.6	5.7
11	5F-MDMB-PICA metabolite 2	1-250	0.25	0.83	103.7	97.5	103.6	67.9	128.2	127.6	2.3	8.8	7.1
12	PX-2	5-250	0.31	1.04	95.6	124.2	104.1	112.5	86.1	105.3	14.3	7.3	10.4
13	5F-ADB-PINACA	5-250	0.20	0.66	125.6	109.4	97.8	122.6	84.9	104.8	12.8	8.5	11.1
14	ADB-BICA	5-250	0.28	0.92	113.7	134.0	88.3	110.4	97.5	74.2	7.2	12.8	7.1
15	5Cl-AB-PINACA	5-250	0.10	0.34	121.5	138.0	106.3	125.1	67.6	77.0	12.6	10.7	10.3
16	JWH-073 *N*-butanoic acid metabolite	1-250	0.11	0.37	103.3	117.4	98.3	62.3	86.3	89.2	14.5	5.4	8.1
17	5-fluoro MDMB-PICA metabolite 7	5-250	0.34	1.13	72.4	102.4	101.2	65.6	93.2	103.4	10.8	5.3	7.5
18	ADB-BINACA	5-250	0.46	1.50	110.5	101.3	103.8	127.3	77.8	79.4	15.3	8.5	5.1
19	4CN-MDMB-BUTINACA	1-250	0.07	0.22	111.2	98.1	107.7	128.1	92.6	96.8	12.0	3.9	9.2
20	ADB-FUBINACA	5-250	0.62	2.04	104.2	110.9	109.3	110.6	77.1	78.7	15.3	9.6	14.2
21	JWH-018 *N*-pentanoic acid metabolite	1-250	0.20	0.65	99.9	105.9	107.4	69.9	89.2	84.9	12.8	6.4	9.4
22	AMB-FUBINACA metabolite	5-250	0.65	2.13	43.6	116.0	110.5	69.3	96.9	93.4	13.0	12.7	5.1
23	AB-PINACA	1-250	0.29	0.96	104.1	71.9	102.1	86.5	81.6	87.6	10.3	9.1	6.5
24	ADB-BUTINACA	1-250	0.25	0.82	101.4	76.8	113.4	96.2	79.9	86.6	9.2	10.2	12.2
25	4F-MDMB-BUTINACA butanoic acid metabolite	5-250	0.71	2.33	90.2	62.8	102.0	80.2	100.5	74.6	9.7	14.4	12.9
26	4CN-CUMYL-BUTINACA	1-250	0.10	0.32	85.1	86.4	102.3	106.2	80.2	77.2	8.5	9.8	13.4
27	ADB-4en-PINACA	1-250	0.26	0.85	103.3	64.2	98.0	107.5	91.0	91.2	7.1	8.3	12.4
28	4F-MMB-BUTINACA	1-250	0.08	0.27	85.6	70.8	108.6	65.1	81.8	72.0	8.6	13.3	4.5
29	UR-144 *N*-pentanoic acid metabolite	1-250	0.08	0.26	93.2	82.5	120.7	70.0	93.8	100.2	13.8	8.3	14.3
30	CUMYL-THPINACA	1-250	0.28	0.92	63.1	67.3	104.2	63.7	91.8	65.4	3.4	5.4	10.4
31	ADB-PINACA	1-250	0.15	0.48	86.2	95.5	109.9	128.5	86.3	100.9	11.4	11.1	13.2
32	MDMB-4en-PINACA butanoic acid metabolite	5-250	0.87	2.88	83.6	90.4	111.2	69.9	100.9	106.1	10.8	5.7	10.6
33	AB-CHMINACA	1-250	0.22	0.73	89.5	102.3	100.4	122.1	83.7	109.1	12.4	8.1	13.0
34	AMB-FUBINACA	1-250	0.16	0.54	93.2	87.8	102.1	92.6	114.3	71.0	8.7	5.0	6.4
35	1-（4-fluorobutyl）-*N*-（2-phenylpropan-2-yl）- 1*H*-indazole-3-carboxamide	1-250	0.10	0.34	72.6	89.1	104.7	71.4	96.8	66.9	6.0	8.8	10.1
36	4F-ABUTINACA *N*-（4-hydroxybutyl） metabolite	1-250	0.14	0.46	84.4	114.6	99.4	108.8	115.7	116.4	15.1	9.9	14.3
37	5F-CUMYL-PINACA	1-250	0.21	0.68	74.9	95.1	98.9	78.1	99.1	69.8	14.1	7.5	6.6
38	EMB-FUBINACA	1-250	0.18	0.60	92.8	93.9	100.4	112.4	116.9	79.6	14.5	7.8	7.2
39	5F-EDMB-PINACA	1-250	0.07	0.23	98.4	90.4	104.1	106.5	124.4	89.6	13.6	13.8	6.3
40	5Cl-ADB	5-250	0.15	0.49	88.7	94.4	99.2	95.3	122.8	100.6	9.6	6.1	11.1
41	MDMB-BUTINACA	1-250	0.03	0.11	84.5	91.4	100.7	74.7	94.3	61.2	10.2	9.9	6.0
42	MDMB-4en-PINACA	1-250	0.07	0.22	86.9	91.4	78.5	78.6	102.8	66.4	10.8	13.7	6.4
43	5F-MN-18	1-250	0.08	0.28	79.6	94.7	78.7	108.3	104.5	99.3	15.5	14.7	11.8
44	5Cl-CUMYL-PINACA	5-250	0.49	1.61	77.2	97.4	101.4	77.5	113.0	77.9	13.8	15.2	11.2
45	4F-ABUTINACA	10-250	0.96	3.15	111.6	106.7	78.3	80.5	120.1	79.6	12.5	12.0	13.8
46	EDMB-PINACA	1-250	0.05	0.18	86.6	98.4	83.2	68.0	111.0	78.3	4.7	13.3	11.8
47	5F-APINACA	5-250	0.49	1.62	85.2	111.3	92.9	67.7	122.0	127.2	2.5	8.3	7.3
48	MDMB-CHMINACA	10-250	0.91	3.02	88.6	127.5	85.8	125.3	116.0	115.0	6.5	10.5	5.6
49	MN-18	1-250	0.07	0.23	92.8	117.3	102.0	67.6	90.6	88.3	10.8	11.2	8.4
50	FUB-APINACA	10-250	0.26	0.86	83.2	108.4	128.3	108.6	111.7	117.0	13.7	10.4	11.3
51	5Cl-APINACA	5-250	0.44	1.45	98.0	75.8	106.3	63.0	87.5	129.3	15.5	2.1	14.2
52	APINACA	1-250	0.20	0.65	88.8	102.5	102.0	66.2	112.9	113.8	6.6	13.3	13.1
53	ACHMINACA	1-250	0.44	1.45	113.9	87.8	100.6	128.0	105.8	128.2	13.6	2.6	5.7

### 2.5 方法学评价

#### 2.5.1 线性关系、检出限与定量限

向纯水中添加38种合成大麻素及15种代谢产物的混合标准溶液，配制成不同质量浓度的系列混合标准溶液进行测定。以各组分的质量浓度与内标的质量浓度之比为横坐标，峰面积之比为纵坐标绘制曲线，曲线采用线性拟合。结果显示，53种目标物在各自的线性范围内线性关系良好，相关系数*r*为0.990 5～0.999 8。分别以信噪比（*S/N*）=3和*S/N=*10确定检出限（LOD）和定量限（LOQ）^［36-38］^。结果显示，53种目标物的检出限为0.03～1.30 ng/L，定量限为0.11～4.30 ng/L（见表2），表示该方法具有较高的灵敏度，可以满足污水中合成大麻素和代谢产物的日常检测需求。

#### 2.5.2 加标回收率

向不含目标物的空白污水样品中添加38种合成大麻素及15种代谢产物的混合标准溶液，在低（10 ng/L）、中（50 ng/L）、高（200 ng/L）3个水平下进行加标回收试验，每个加标水平平行测定6次，计算回收率。计算得出，各目标物的加标回收率为61.2%～129.3%，表明该方法的定量分析结果具有准确性。53种目标物的加标回收率见表2。

#### 2.5.3 精密度

向不含目标物的空白污水样品中添加38种合成大麻素及15种代谢产物的混合标准溶液，使53种目标物的质量浓度分别为10、50、200 ng/L。每个水平平行测定6次，计算得到精密度（RSD），相关数据见表2。由表2可知，53种目标物的精密度为2.1%～15.5%，说明该方法的精密度良好。

### 2.6 方法对比

将本文中所建立的方法与其他文章中污水中合成大麻素及代谢物的检测方法进行对比，如表3所示。本方法针对的是吲唑酰胺类合成大麻素及代谢产物，检测的合成大麻素种类较新，更有针对性，且数量较多。此外，本方法检测效率较高，检出限和定量限与其他方法相比，均处于较低水平，能够满足日常检测的需求。

**表3:** 本方法与其他方法的比较

Actual sample	Numbers of target compounds	Detection method	Instrumental analysis time/min	LOD/（ng/L）	LOQ/（ng/L）	Ref.
Wastewater	38 indazole amides SCs and 15 metabolites	LC-MS/MS	18	0.03-1.30	0.11-4.30	this study
Wastewater	8 SCs and 8 metabolites	LC-MS/MS	12.5	0.005-0.02	0.04-0.25	［^26^］
Wastewater	9 SCs	LC-MS/MS	11	0.23-10	0.70-32	［^27^］
Wastewater	7 SCs	LC-HRMS	30	2.0-23.6	-	［^29^］
Wastewater	5 SCs and 2 metabolites	LC-HRMS	18	-	-	［^30^］
Wastewater	4 metabolites	UHPSFC-MS/MS	6	-	1.0-6.5	［^39^］
Wastewater	5 SCs	UHPLC-MS/MS	6	-	0.1-1.0	［^40^］
Wastewater	11 SCs	UHPLC-MS/MS	<26	0.1-6.3	0.4-21.0	［^41^］
Wastewater	13 SCs and 11 metabolites	LC-MS/MS	12.1	5-500	100-10000	［^42^］

UHPSFC： ultra high performance supercritical fluid chromatography.

### 2.7 实际样品检测

利用该方法对10份某省不同污水处理厂进水进行38种吲唑酰胺类合成大麻素及15种代谢物质的检测分析。结果表明，在10份污水样品中均未检出该方法中所包含的合成大麻素类物质及代谢产物。

## 3 结论

本文建立了固相萃取-液相色谱-三重四极杆质谱法（LC-MS/MS）同时检测城市生活污水中38种吲唑酰胺类合成大麻素及15种代谢产物的方法。该方法可同时对38种吲唑酰胺类合成大麻素及15种代谢产物进行定性和定量分析。该方法高效简便，检测目标物的数量较多，准确度和精密度良好，适用于城市生活污水中合成大麻素类物质及代谢产物的检测分析。应用该方法对城市生活污水中的合成大麻素及代谢产物进行监测，可以有效对合成大麻素类物质的滥用情况进行预警分析，具有较高的实用性，对于实际执法具有重要意义。
